# RNA variation as the driver of genomic efficiency and phenotypic complexity

**DOI:** 10.7150/ijbs.135200

**Published:** 2026-05-29

**Authors:** Zhihua Jiang, Michee van Rooyen, Jennifer J. Michal, Isyana Khaerunnisa, Saiful Anwar, Xing Fu, Jinzeng Yang, Steve P. Miller

**Affiliations:** 1 Department of Animal Sciences, Washington State University, Pullman, WA, USA.; 2 Research Center for Applied Zoology, Research Organization for Life Sciences and Environment, National Research and Innovation Agency, Republic of Indonesia.; 3 School of Animal Sciences, Louisiana State University, Baton Rouge, LA, USA.; 4 Department of Human Nutrition, Food and Animal Sciences, University of Hawaii at Manoa, Honolulu, HI, USA.; 5 Animal Breeding and Genetic Unit, University of New England, Armidale, NSW 2351, Australia.

**Keywords:** RNA variation, gene biotypes, alternative processing, RNA editing and modification, transcript diversity, genomic efficiency

## Abstract

Despite decades of genome sequencing and annotation, a fundamental paradox remains unresolved: how organisms with finite and relatively stable gene numbers generate extraordinary phenotypic diversity. In this review, we propose that regulated RNA variant diversity provides a critical level of resolution for understanding genome expression, which we describe as a conceptual “minimal functional unit” for distinguishing distinct regulatory or functional outcomes. We synthesize evidence that transcript diversity arises through hierarchical and combinatorial RNA variation operating at three levels. At the genome level, diverse gene biotypes—including protein-coding genes, non-coding RNAs, pseudogenes, transposable elements, and bifunctional loci—expand regulatory capacity beyond classical gene definitions. At the gene level, alternative transcription initiation, splicing, and polyadenylation generate structurally and functionally distinct transcript isoforms. At the transcript level, RNA editing and chemical RNA modifications further modulate RNA stability, localization, translation, and immune recognition, forming a dynamic epitranscriptome. Together, these layers of RNA variation dramatically amplify functional output without increasing gene number, providing a coherent framework for linking static genomes to dynamic phenomes. By integrating conceptual models and biological examples, this review highlights RNA variation as a central organizing principle in genetics, with broad implications for evolution, development, and disease.

## 1. Introduction

**Figure 1:**
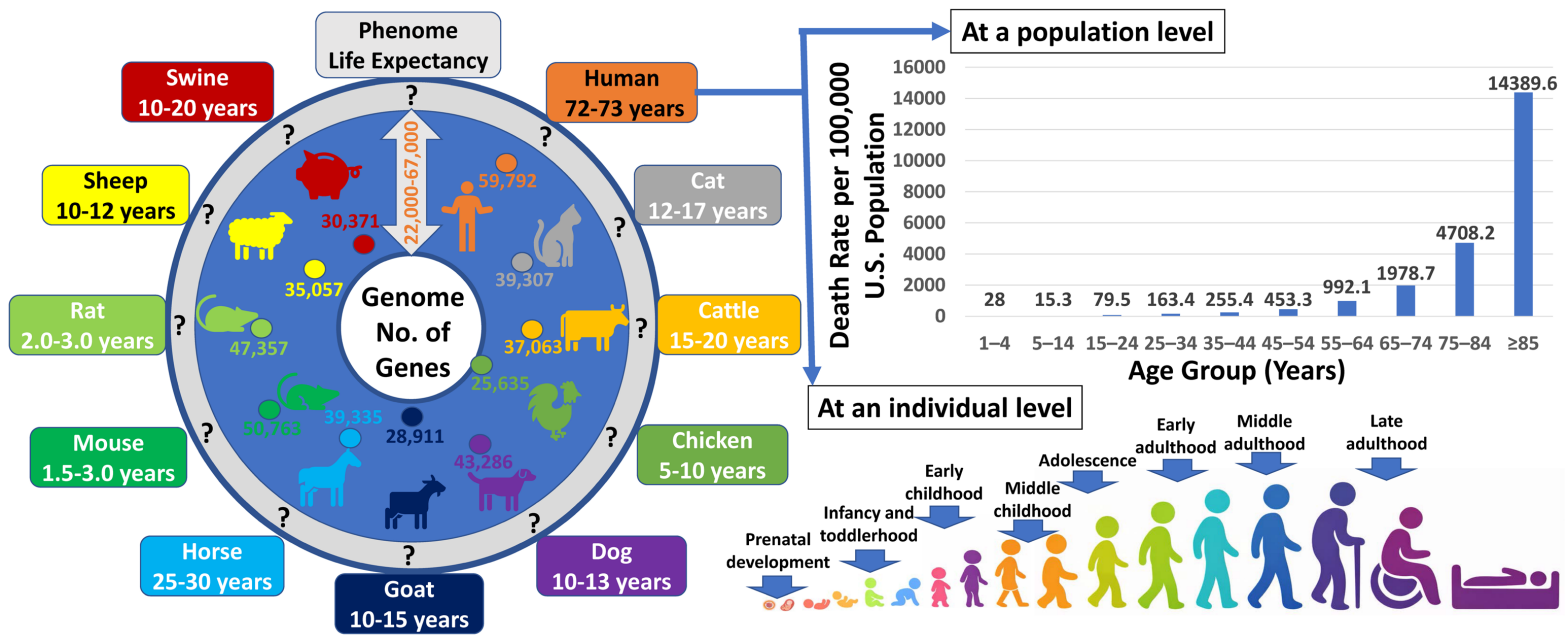
The complex relationship between genome and phenome. Across organisms, the number of annotated genes per genome falls within a surprisingly narrow range (approximately 22,000-67,000), a scale already anticipated by early studies in 1948 1 and 1964 2, despite the absence of genome sequence data at the time. In contrast, phenotypic outcomes vary dramatically across species and within populations. Mammalian and avian phenotypes unfold over lifespans ranging from approximately 1.5-3 years in mice to 72-73 years in humans. Even within human populations, life expectancy can range from minutes after birth to more than 100 years, despite genomes sharing ~99.9% sequence identity. Moreover, an individual's phenotypes continuously change over the course of a lifetime while the underlying genome remains largely constant. Together, these observations highlight the profound complexity of genome-phenome relationships and underscore the need for regulatory frameworks beyond gene number alone.

The completion of numerous genome sequencing projects revealed a striking and enduring paradox: organisms of vastly different complexity possess surprisingly similar numbers of genes (**Figure 1**). From invertebrates to mammals, gene counts vary far less than phenotypic diversity, challenging the long-held assumption that biological complexity scales with gene number 1-5. This discrepancy has shifted attention from gene catalogues to regulatory mechanisms that shape how genomic information is deployed 6-8. Yet even regulatory explanations remain incomplete unless the functional outputs of genes are considered explicitly. In this review, we argue that regulated RNA variants provide a critical level of resolution for understanding gene function, as they represent the most proximal molecular level at which genomic information is realized. We use the term “minimal functional units” to denote a conceptual level of transcript resolution at which distinct regulatory or functional outcomes can be distinguished, offering a framework for understanding how finite genomes generate dynamic, context-dependent phenotypes 9,10.

Early efforts to resolve the gene number paradox emphasized transcriptional and chromatin-level regulation, highlighting cis-regulatory elements, chromatin accessibility, and developmental transcription programs as key drivers of phenotypic diversity 11-13. While these mechanisms shape gene activity, they do not fully account for how identical genes produce distinct functional outputs across tissues, developmental stages, sexes, and environmental conditions 14-15. Gene-level regulation determines whether a locus is active, but it does not specify the precise molecular forms through which genetic information is ultimately realized. This distinction becomes critical when considering that a single gene can give rise to multiple RNA molecules with divergent regulatory, structural, and functional properties 16,17.

Transcriptome-wide analyses have revealed that RNA diversity is pervasive rather than exceptional. Most genes generate multiple RNA variants through alternative transcription start site usage, alternative exon splicing, and alternative polyadenylation 18-20. These processes do not operate independently but are often coordinated, producing transcript isoforms that differ in untranslated regions, coding potential, stability, localization, and translational efficiency. Importantly, such variation can alter cellular behavior without changing protein sequence, underscoring the functional significance of transcript architecture itself.

Beyond RNA processing, additional layers of diversity arise from the expanding repertoire of RNA species encoded within genomes. Protein-coding genes coexist with long and short non-coding RNAs, pseudogenes, transposable element-derived transcripts, and bifunctional loci capable of both coding and regulatory roles 3,4,7,8. These diverse gene biotypes contribute to regulatory networks that operate primarily at the RNA level, blurring traditional distinctions between genes and regulatory elements. Together, they expand the functional landscape of the genome without a proportional increase in gene number.

Further complexity is introduced by chemical modification and editing of RNA molecules. Specifically, more than one hundred distinct RNA modifications have been identified across coding and non-coding transcripts, influencing RNA folding, stability, localization, translation, and immune recognition 9,10,19. This epitranscriptomic layer is dynamic and context dependent, enabling rapid modulation of RNA behavior in response to developmental cues and environmental stimuli. Importantly, RNA modifications often act in concert with alternative RNA processing, further diversifying transcript function beyond what can be inferred from sequence alone.

Taken together, these observations point to a conceptual shift in how genome function is understood. Rather than viewing genes as singular functional entities, it is increasingly clear that regulated RNA variants constitute the operational units through which genetic information is executed. RNA variation integrates signals from genome architecture, transcriptional regulation, post-transcriptional processing, and chemical modification to generate a flexible and scalable system of biological control. This framework provides a parsimonious explanation for how finite genomes achieve regulatory depth, phenotypic plasticity, and evolutionary adaptability 5,6,14,15,20.

In this review, we synthesize evidence supporting an RNA-variant-centric view of genome function. We first examine RNA variation at the genome level, focusing on the contribution of diverse gene biotypes to regulatory capacity. We then discuss how coordinated alternative transcription initiation, splicing, and polyadenylation generate functionally distinct transcript isoforms from individual loci. Finally, we consider how RNA modifications and editing further refine transcript fate and function. By integrating these layers, we propose RNA variation as a unifying principle that links static genomes to dynamic phenomes in development, evolution, and disease 7-10,12,13,17,18.

## 2. RNA variation at the genome level

**Figure 2:**
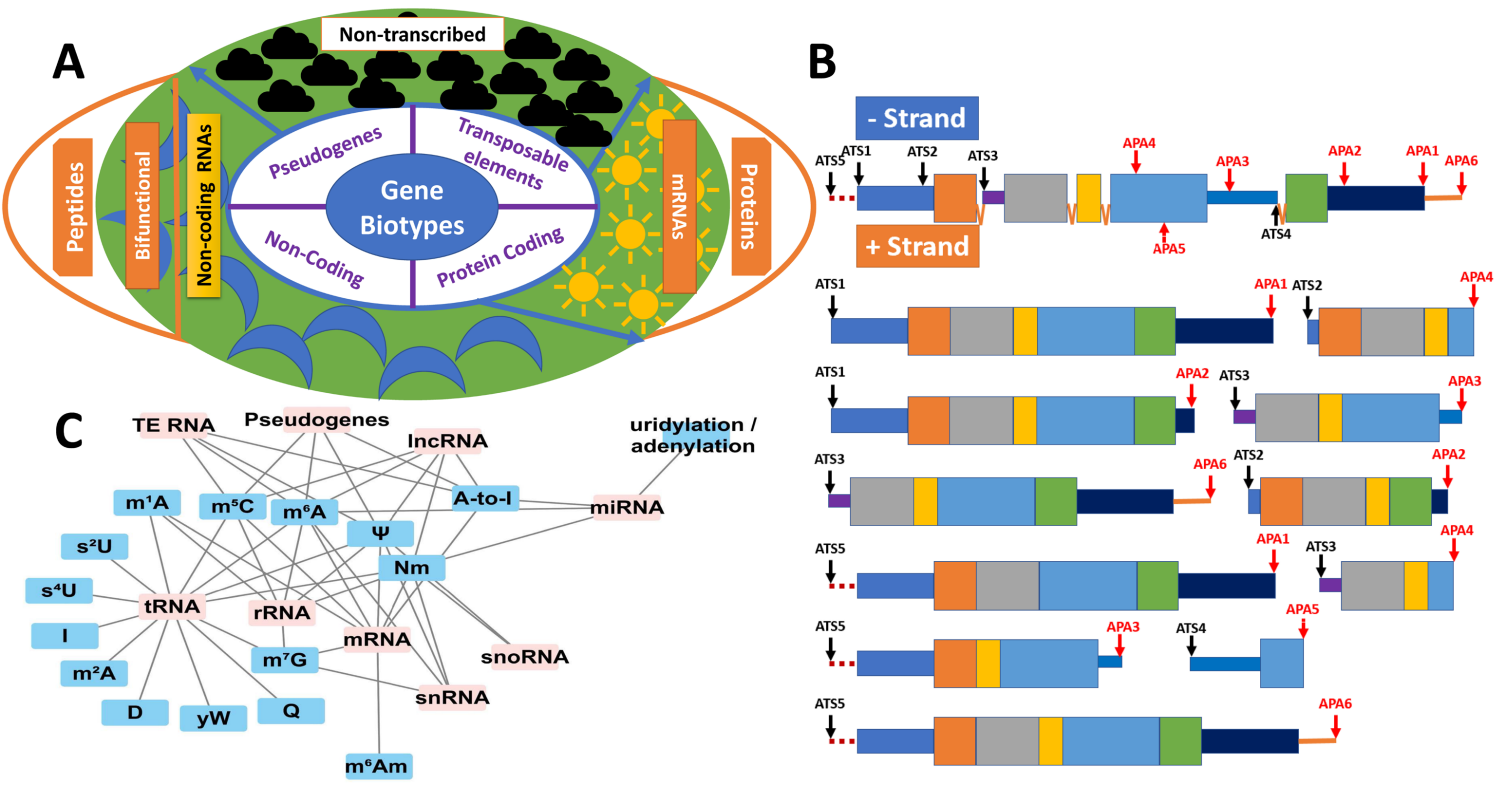
The genome as a generator of extensive RNA variation. (A) RNA variation at the genome level. Genes can be broadly classified into four categories. Protein-coding genes produce both mRNAs for translation into proteins and non-coding RNA isoforms. Non-coding RNA genes generate regulatory RNAs as well as bifunctional RNAs with peptide-coding potential. Pseudogenes may be transcriptionally active or inactive; when transcribed, their RNAs can function as non-coding or bifunctional molecules. Transposable elements may also be transcriptionally active or silent, contributing additional sources of RNA variation when expressed.(B) RNA variation at the gene level. A representative gene containing five exons (with the first exon harboring a 5′ untranslated region (UTR) and the last exon a 3′ UTR), five alternative transcription start (ATS) sites, and six alternative polyadenylation (APA) sites illustrates the diversity of transcripts that can be produced from both the positive and negative strands. Partial intron retention (introns 1 and 4) further expands transcript diversity. ATS and APA sites can extend upstream of canonical start sites or downstream of annotated transcriptional termini. (C) RNA variation at the transcript level. Distinct classes of RNA exhibit characteristic patterns of chemical modification, adding an additional regulatory layer that influences RNA stability, localization, and function.

At the genomic level, genes are generally classified into four main categories: protein-coding genes, non-coding genes, pseudogenes, and other types of genes (**Supplemental Table S1** and **Figure 2A**). According to the current human genome annotation (GRCh38.p14), there are 59,792 genes in total, including 20,076 protein-coding genes, 17,066 pseudogenes, 22,179 non-coding genes, 399 immunoglobulin/T-cell receptor gene segments, and 72 other genes. In addition to these gene types, genomes contain a significant number of transposable elements (TEs), or “jumping genes.” These mobile genetic elements, which make up at least 45% of the human genome, are distributed across the genome—in genes, between genes, and in non-coding regions—and play critical roles in gene regulation, genome evolution, and genetic diversity 21.

In this review, we will explore five key aspects of genome transcription: 1) protein-coding genes in translation and beyond; 2) non-coding RNA genes in functional regulation; 3) pseudogenes in competing endogenous RNA networks; 4) transposable elements in shaping RNA diversity; and 5) bifunctional non-coding genes encoding micropeptides. Together, these aspects illustrate how the diversity of RNA biotypes and their functions drive the dynamic complexity of the transcriptome, shaping gene regulation, cellular activity, and phenotypic diversity beyond the constraints of the finite genome.

### 2.1. Protein-coding genes in translation and beyond

Protein-coding genes primarily give rise to messenger RNAs (mRNAs), which are translated into proteins, the fundamental building blocks of life 22. Proteins perform a wide range of essential biological functions, including catalysis, immunity, metabolism, movement, protection, regulation, storage, structure, transport, and much more 23.

Many protein-coding genes are conserved across species and are referred to as orthologous genes 24. A subset of these genes also function as hosts for embedded non-coding RNA (ncRNA) genes 25. Notably, microRNAs (miRNAs) and small nucleolar RNAs (snoRNAs) are frequently found within such host genes. In the human genome, more than 75% of these embedded ncRNAs are transcribed from the same strand as their host gene, most often residing within introns 25. A single intron can contain one or multiple ncRNAs, which may be co-transcribed alongside the host gene or expressed independently, depending on their biotype 25.

Interestingly, in addition to producing mRNAs, some protein-coding genes can also encode for ncRNAs. These are known as bifunctional protein-coding genes and can arise through three main mechanisms 26. First, some long noncoding RNAs (lncRNAs) contain small open reading frames (sORFs) that are translated into functional micropeptides 26. Second, certain mRNAs perform additional noncoding roles—such as regulating transcription, translation, or acting as scaffolds—independent of their protein products 26. Third, alternative splicing or other regulatory processes can generate noncoding isoforms from protein-coding genes, producing stable transcripts with functional roles beyond protein synthesis 26.

Bifunctionality can also result in allele-specific DNA variants 26,27. Mutations in promoter and enhancer regions may lead to the production of non-coding transcripts or loss of protein-coding transcripts 27. Similarly, mutations such as insertions/deletions, frameshifts, gain of stop codons, loss of start/stop codons, or changes of splicing donor/acceptor sites can disrupt the protein-coding functions 27. These variants may also alter RNA-binding sites, affecting interactions with RNA-regulatory factors and leading to the production of alternative transcript isoforms, including non-coding RNA.

The third arises from the usage of alternative transcription start sites, splicing, or polyadenylation sites, resulting in the production of long non-coding RNAs (lncRNAs), which are typically ≥ 200 bp in length 28,29. These lncRNAs can be classified into six types based on their genomic location and relationship to protein-coding genes: intronic lncRNAs, exonic lncRNAs, antisense lncRNAs, sense lncRNAs, bi-directional lncRNAs, and circular lncRNAs 28,29. We will delve into the functional significance of these lncRNAs in Section 3: RNA variation at the gene level.

### 2.2. Non-coding RNA genes in functional regulation

Non-coding genes are transcribed into ncRNAs, which are not translated into proteins 30,31. Based on their expression, function, and structural features, ncRNAs are broadly classified into two groups: housekeeping and regulatory.

Housekeeping ncRNAs are ubiquitously expressed in most, if not all, cell types. Currently, five major types of non-coding RNA genes that produce housekeeping RNAs have been identified, including ribosomal RNAs (rRNAs), transfer RNAs (tRNAs), small nuclear RNAs (snRNAs), small nucleolar RNAs (snoRNAs), and telomerase RNAs (teRNAs). These ncRNAs play fundamental roles in maintaining cell viability and function, including protein synthesis (rRNA and tRNA), RNA splicing (snRNAs), RNA modification (snoRNAs), and genome integrity (teRNAs).

Regulatory non-coding RNAs, also known as "riboregulators," play fundamental roles in gene expression. They modulate chromatin structure, regulate transcription and translation, influence protein activity, and control the localization of proteins and RNA within cells. These ncRNAs are generally categorized by length. Small non-coding RNAs, generally <200 bp in length, include a diverse assortment of molecules such as microRNA (miRNA), miRNA-offset RNA (moRNA), short hairpin-derived miRNA (shRNA-derived miRNA), miRNA-like small RNA (milRNA), Piwi-interacting RNA (piRNA), endogenous small interfering RNA (endo-siRNA), tRNA-derived RNA fragment (tRF), tRNA-derived small RNA (tsRNA), promoter-associated small RNA (PASR), termini-associated small RNA (TASR), transcription start site-associated RNA (TSSa-RNA), transcription initiation RNA (tiRNA), splice-site RNA (spliRNA), snoRNA-derived RNA (sdRNA), QDE-2-interacting small RNA (qiRNA) and small vault RNA (svRNA), for example 32. Among these, miRNAs and piRNAs are the most extensively studied small ncRNAs due to their abundance, evolutionary conservation, and central roles in gene regulation. miRNAs broadly control mRNA stability and translation in diverse biological processes and diseases, while piRNAs safeguard genome integrity in the germline by silencing transposable elements 32.

In contrast, lncRNAs are typically ≥200 bp in length. While 6 types of lncRNAs associated with protein-coding genes have been discussed previously, this section focuses on long intergenic non-coding transcripts (lincRNAs), which account for approximately 50% of all lncRNAs 33. LincRNAs genes are located between protein-coding genes and do not overlap with them 34,35. Compared to mRNAs, lincRNAs are generally shorter, contain fewer exons, and are expressed at lower levels. They are more frequently located in the nucleus than in the cytoplasm. Like mRNAs, lincRNAs can exhibit tissue-specific and time-sensitive expression patterns. Interestingly, they may function as enhancers, regulating the expression of nearby genes 34,35. However, lincRNAs are typically not evolutionarily conserved, making it hard to identify orthologs across species.

### 2.3. Pseudogenes in competing endogenous RNA networks

Pseudogenes are genomic elements that resemble functional genes but have lost protein-coding ability due to accumulated mutations or structural disruptions. Once viewed as non-functional evolutionary relics, they are now recognized as important components of regulatory gene networks 36. Pseudogenes typically arise through gene duplication or retrotransposition and, despite lacking protein-coding capacity, their transcripts can modulate gene expression and influence biological processes such as regulation, differentiation, and responses to environmental stimuli 36.

Based on their origin and structure, pseudogenes fall into three categories 36. Processed pseudogenes originate from retrotransposed mRNA lacking introns and regulatory elements and are often not expressed, though their sequences can still interact with other RNAs. Unprocessed (duplicate) pseudogenes arise from gene duplication followed by disabling mutations and retain introns as well as regulatory regions that may allow transcription. Unitary pseudogenes result from the inactivation of single-copy genes and lack functional counterparts in the genome. These categories illustrate the diverse evolutionary pathways leading to pseudogene formation.

Although pseudogenes cannot encode proteins, many are transcribed into RNA, revealing functions beyond passive genomic remnants 37-40. Pseudogene-derived long non-coding RNAs (lncRNAs) are increasingly recognized for their regulatory roles, particularly in cancer where they frequently act as competing endogenous RNAs (ceRNAs) 39. By sequestering microRNAs (miRNAs), these transcripts can relieve repression of miRNA-targeted genes; for example, PTENP1 modulates expression of the tumor suppressor PTEN, contributing to cancer progression 40. Such ceRNA activity highlights the capacity of pseudogenes to shape gene regulatory networks.

Beyond cancer, pseudogene dysregulation contributes to a wide range of human diseases. In cardiovascular disease, pseudogene transcripts influence processes underlying atherosclerosis, myocardial infarction, fibrosis, cardiomyopathy, pulmonary arterial hypertension, and aortic aneurysm or dissection through effects on TGF-β1 signaling, PI3K-AKT pathways, smooth muscle apoptosis, and vascular remodeling 41. Pseudogenes are also implicated in neurodegenerative disorders, including Alzheimer's, Parkinson's, and Huntington's disease, where ceRNA-mediated shifts in miRNA availability disrupt pathways commonly altered in cancer, such as apoptosis, p53, Ras, PDGF, FGF, EGF, and MAPK signaling 42. Together, these findings underscore that pseudogenes play active—and sometimes pathogenic—roles across diverse diseases by reshaping post-transcriptional regulatory networks.

### 2.4. Transposable elements in shaping RNA diversity

Transposable elements (TEs) are mobile DNA sequences that occupy a large portion of the vertebrate genomes and play a critical role in genome evolution and regulation. TEs include retrotransposons (such as LINE-1, SINEs/Alu, SVA, endogenous retroviruses) and DNA transposons in human. Although most TE copies have lost the capacity for active transposition, many remain transcriptionally competent under certain conditions 43,44. TE transcription is normally suppressed in somatic cells by multilayered epigenetic mechanisms — including DNA methylation, repressive histone modifications mediated by KRAB-ZFP/KAP1, histone deacetylases, and RNA-based silencing — which preserve genomic stability 45,46. However, during key biological contexts such as early development, cellular differentiation, or stress, repression can be relaxed, allowing TEs to be transcribed. TE-derived RNAs can arise from autonomous TE promoters or result from read-through transcription of host genes, thereby contributing to gene regulatory variation, chromatin remodeling, immune activation, and cellular plasticity 47.

Methods such as TEtranscripts 48, SQuIRE 49, long-read RNA-seq-based TE transcript annotation frameworks 50, and TE-Seq 51 collectively address the central challenge of identifying and interpreting TE expression from sequencing data. Because TE-derived reads are often repetitive and ambiguously mapped, TEtranscripts improves detection by probabilistically assigning multi-mapping reads and incorporating them into differential expression analyses alongside genes, thereby recovering TE signals that are typically overlooked 48. SQuIRE advances this further by resolving TE expression at the locus level rather than aggregating signals across subfamilies, enabling interpretation of genomic context, regulatory relationships, and splicing patterns that shape TE activity 49. Complementing short-read approaches, long-read RNA-seq-based annotation pipelines provide full-length transcript resolution, allowing the discovery of novel TE-derived transcripts, isoforms, and TE-gene fusion events, as well as cell-type-specific expression patterns 50. Finally, TE-Seq integrates these concepts into an end-to-end workflow that quantifies TE expression across both reference and non-reference loci and at multiple levels (individual elements and clades), facilitating biological interpretation across conditions such as development or disease 51. Together, these methods enable more accurate quantification, locus-specific resolution, and functional interpretation of TE transcriptional activity in complex transcriptomes.

These methodological advances have revealed that TE-derived transcripts are not silent artifacts but active players with functional consequences in physiology and disease. In normal biology, TEs contribute to gene regulation by supplying alternative promoters, enhancers, splice sites, and polyadenylation signals, thereby shaping transcriptional networks, chromatin architecture, and cell identity — especially during early development, immune responses, and cell differentiation. TE RNAs can serve as regulatory non-coding RNAs, scaffold chromatin modifiers, or produce chimeric transcripts that influence nearby genes 47,49. In disease, aberrant TE activation — often due to epigenetic derepression — is associated with genomic instability, insertional mutagenesis, dysregulated gene expression, chronic inflammation, and immune activation 46,47,52. For example, increased TE expression in tumors can elevate the expression of oncogenes or suppress tumor suppressors, contribute to structural genome variation, or produce TE-derived neoantigens that stimulate immune responses, making TEs relevant for both cancer diagnosis and therapy 47,48. Similarly, TE dysregulation has been linked to neurodegenerative disorders, autoimmune diseases, and age-associated pathologies, where TE RNAs may trigger innate immune sensors, alter gene regulation, or impair cellular homeostasis 47,52.

### 2.5. Bifunctional non-coding genes encoding micropeptides

The classical view of the transcriptome distinguishes protein-coding mRNAs from non-coding RNAs (ncRNAs). However, advances in ribosome profiling, proteogenomics, and computational ORF prediction have revealed a growing class of bifunctional non-coding genes. These transcripts—often annotated as long non-coding RNAs (lncRNAs)—harbor small open reading frames (sORFs) capable of producing biologically active micropeptides, usually fewer than 100 amino acids in length. These bifunctional molecules retain RNA-level regulatory properties while also contributing to the proteome, thereby challenging long-standing assumptions about RNA function and genome annotation 53,54.

Micropeptides encoded by bifunctional lncRNAs participate in diverse cellular processes, including signal transduction, metabolic regulation, mitochondrial function, genome stability, and cell differentiation 55-57. Several have demonstrated major roles in human disease. For example, the lncRNA *LINC00961* encodes the micropeptide SPAAR, which regulates endothelial cell function and muscle regeneration 55. *HOXB-AS3* produces a 53-aa tumor-suppressor peptide that inhibits glycolysis in colorectal cancer 56. *LINC00675* encodes FORCP, a micropeptide that suppresses colon cancer proliferation 57. These examples illustrate that bifunctional lncRNAs expand functional diversity and offer new opportunities for therapeutic targeting.

Discovery of peptide-coding lncRNAs requires integration of dry-lab (computational) and wet-lab (experimental) approaches. Ribosome profiling (Ribo-seq) provides high-resolution maps of ribosome-protected fragments, revealing active translation on transcripts previously labeled non-coding 58. Complementary computational tools—including CPC2**,** PhyloCSF**,** ORFfinder, and machine-learning-based sORF detectors—evaluate coding potential using ORF length, codon usage, evolutionary conservation, and Kozak sequence context 59,60. Large-scale databases such as sORFs.org and TransLnc integrate ribosome profiling and proteomics to catalog thousands of predicted micropeptides 61,62.

Experimental validation is essential because many sORFs lack evolutionary conservation and may produce unstable or low-abundance peptides. Mass spectrometry (MS) remains the gold standard for micropeptide detection and sequencing 63. Additional methods such as epitope-tagged ORF expression, CRISPR-mediated ORF disruption, reporter assays, and cross-species conservation analyses are widely used to confirm translation and determine physiological function 64. Together, these methods distinguish true bifunctional lncRNAs from non-coding transcripts with spurious ribosome association.

Micropeptides derived from lncRNAs have increasingly recognized roles in development, metabolism, immunity, and disease. In cancer, bifunctional lncRNAs can act as oncogenes or tumor suppressors, modulating pathways such as PI3K-AKT, mTORC1, MAPK, or metabolic reprogramming 56,57,60. In addition to cancer biology, micropeptides contribute to muscle development, cardiac physiology, mitochondrial homeostasis, and genome stability. The lncRNA *NORAD*, for example, produces peptides that help maintain chromosomal stability by interacting with PUMILIO proteins; loss of this function leads to aneuploidy and contributes to cancer and age-related disorders 65. Micropeptides such as SMIM30, encoded by *LINC00998*, regulate oncogenic kinase signaling in liver cancer and correlate with poor patient prognosis 66. Collectively, bifunctional lncRNAs reveal an additional layer of gene regulation with profound implications for biomarker discovery, therapeutic development, and personalized medicine.

## 3. RNA Variation at the Gene Level

RNA variation at the gene level arises from diverse post-transcriptional processes that expand the functional output of the genome (**Supplemental Table S2** and **Figure 2B**). Key mechanisms include alternative transcription start sites (ATS), alternative exon splicing (AES), and alternative polyadenylation (APA), which together enable a single gene to produce multiple RNA isoforms with distinct structures and regulatory properties 67-72. ATS occurs when a gene harbors multiple transcription initiation sites, generating transcripts with variable 5' leader sequences that influence stability, translation efficiency, and protein-coding potential 67,71. AES modifies pre-mRNA by selectively including or excluding exons, producing mRNAs with differing coding sequences or untranslated regions, thereby affecting localization, stability, and translation 67-69. APA involves the use of alternative polyadenylation sites, yielding transcripts with variable 3' ends that further regulate mRNA stability, translation, and cellular localization 67,70,73. Here we would focus our review on 1) interconnected regulation among ATS, AES and APA sites; 2) isoforms of the same gene differ in RNA stability, localization, and translation; and 3) alternative transcripts can be cell-, tissue-, or time-specific.

### 3.1. Interconnected regulation among ATS, AES and APA events

While dysregulation of RNA processing can contribute to disease, alternative RNA processing mechanisms are fundamental to normal gene expression. Many genes naturally generate multiple RNA isoforms through alternative transcription start site usage (ATS), alternative exon splicing (AES), and alternative polyadenylation (APA). Genome-wide studies have shown that more than half of human genes utilize ATS, approximately 70% employ multiple polyadenylation sites, and over 95% undergo alternative splicing, often in tissue-specific, developmental stage-specific, or signal-dependent contexts 70,71,74. These mechanisms substantially expand transcriptomic complexity beyond what is predicted by gene number alone 69.

Together, ATS, AES, and APA form a coordinated regulatory network that enables a single gene to produce transcript isoforms with distinct coding potential, stability, translational efficiency, and subcellular localization 69. Most mammalian genes leverage multiple RNA processing mechanisms simultaneously, creating diverse isoform repertoires that support cellular specialization and developmental plasticity 69,70. This coordinated regulation allows organisms to achieve remarkable biological complexity despite a limited number of protein-coding genes 74.

Importantly, these mechanisms do not operate independently but are functionally and mechanistically coupled. Promoter choice and ATS can influence downstream splicing decisions through promoter-dependent recruitment of transcription factors, chromatin modifiers, and splicing regulators, as well as through changes in RNA polymerase II elongation kinetics 75,76. Chromatin structure and histone modifications associated with alternative promoters further shape splice site recognition 77. In parallel, AES and APA are tightly interconnected, as splicing factors can regulate polyadenylation site choice and components of the cleavage and polyadenylation machinery can influence splice site selection, particularly in terminal exons 71,76.

Collectively, these findings demonstrate that transcription initiation, splicing, and polyadenylation function as an integrated, co-transcriptional system rather than as isolated processing steps 75,76. The transcriptional and chromatin context of a gene shapes the pre-mRNA substrate and guides coordinated isoform selection across the gene body. This integrated regulation ensures transcriptome flexibility while maintaining precise control over gene expression outcomes. Disruption of this coordination contributes to numerous pathologies, including cancer and other complex diseases 78.

### 3.2. Isoforms of the same gene differ in RNA stability, localization, and translation

RNA isoforms produced from the same gene can exhibit substantial differences in stability, subcellular localization, and translational efficiency, even when encoding identical protein sequences. These functional differences are largely driven by variation in untranslated regions (UTRs), sequence motifs, and RNA structural features generated through alternative transcription start site (ATS) usage and alternative polyadenylation (APA). As a result, RNA isoform diversity adds an additional regulatory layer that shapes gene expression outcomes beyond protein coding potential 79,80.

RNA stability is strongly influenced by the length and composition of the 3′ untranslated region (3′ UTR), RNA secondary structure, and the presence or absence of binding sites for microRNAs (miRNAs) and RNA-binding proteins (RBPs) 71,79,80. APA plays a central role in this process by generating isoforms with different 3′ UTR lengths. Isoforms using proximal polyadenylation sites often have shorter 3′ UTRs that lack destabilizing elements such as AU-rich elements or miRNA binding sites, rendering them more stable and less susceptible to degradation. In contrast, isoforms with longer 3′ UTRs are more frequently targeted by RBPs and miRNAs that promote mRNA decay, leading to reduced transcript half-life and lower protein output 79-81.

Even closely related isoforms differing only at their 3′ ends can show marked differences in stability and translational capacity 82,83. These differences are partly mediated by poly(A)-binding protein (PABP) interactions, which protect transcripts from exonucleolytic decay and promote translation initiation. The efficiency of PABP binding depends on the precise sequence and structural context of the 3′ end, directly linking APA site choice to mRNA persistence and translational efficiency 82,83. Thus, APA not only governs transcript abundance but also coordinates mRNA stability with protein production.

Subcellular localization of RNA isoforms represents another critical functional consequence of alternative RNA processing. Localization signals embedded within both the 5′ and 3′ UTRs direct mRNAs to specific cellular compartments, thereby controlling where translation occurs 84,85. ATS and APA can generate isoforms that include or exclude these localization elements, enabling spatially restricted gene expression. Longer 3′ UTRs frequently harbor localization signals that target transcripts to specialized regions such as neuronal synapses or the leading edge of migrating cells, whereas shorter isoforms lacking these signals tend to remain diffusely distributed in the cytoplasm or near the nucleus 84-86.

Translational efficiency is also strongly modulated by UTR architecture. The 5′ UTR regulates ribosome recruitment and scanning through features such as upstream open reading frames (uORFs) and RNA secondary structures, including stem-loops and G-quadruplexes. Isoforms generated by alternative ATS usage can differ substantially in 5′ UTR length and structure, leading to pronounced differences in translation initiation efficiency 87,88. In parallel, shorter 3′ UTRs generated by APA often enhance translation by evading miRNA- or RBP-mediated repression, further coupling RNA processing to protein output 71,80. Together, these mechanisms demonstrate how alternative RNA processing expands gene function by modulating RNA fate, localization, and translational control across tissues, developmental stages, and cellular conditions.

### 3.3. Alternative transcripts can be cell-, tissue-, or time-specific

Alternative RNA transcripts exhibit extensive cell-, tissue-, and time-specific variation, which plays a central role in shaping gene expression programs, development, and physiological function. This specificity arises from differential usage of ATS, AES, and APA sites, which together generate context-dependent RNA isoform repertoires. Large-scale transcriptomic analyses demonstrate that isoform diversity often reflects cellular identity more accurately than total gene expression levels, highlighting RNA processing as a key regulatory layer underlying phenotypic complexity 89,90.

The selection of ATS sites varies substantially between tissues and cell types and is a major contributor to context-specific transcript variation. In fact, many transcript differences between human tissues arise from alternative usage of transcription start and termination sites rather than from splicing alone 89,91. Tissue-specific transcription factors and promoter architectures activate distinct ATS sites within the same gene, producing isoforms with variable 5′ untranslated regions (5′ UTRs). These differences directly affect mRNA stability, localization, and translational efficiency; for example, longer 5′ UTRs may contain upstream open reading frames (uORFs) or inhibitory secondary structures, whereas shorter 5′ UTRs often promote more efficient translation. Thus, ATS usage enables fine-tuned, cell-type-specific control of gene expression outputs 87.

AES represents one of the most versatile mechanisms for generating transcript diversity and is tightly linked to tissue identity and developmental timing. Splicing decisions are governed by cis-regulatory RNA elements and trans-acting splicing factors, many of which are expressed in a cell- or tissue-restricted manner. In neurons and muscle cells, for example, alternative splicing generates isoforms essential for specialized cellular functions such as synaptic signaling and contractility 92,93. During embryonic development, global shifts in splicing patterns accompany cell fate transitions, reinforcing the role of AES as both a driver and a consequence of differentiation and tissue maturation 94.

APA further contributes to cell-type and context specificity by regulating 3′ untranslated region (3′ UTR) length and composition. Tissues such as the brain and testis preferentially utilize distal polyadenylation sites, generating longer 3′ UTRs enriched in regulatory elements that mediate RNA localization, stability, and microRNA-dependent control 71,95. APA is also dynamically regulated across developmental and physiological states. In *Xenopus tropicalis*, APA coordinates embryonic development, sexual dimorphism, and growth, while in *Drosophila*, distinct APA patterns are observed not only between tissues but also among individual cell types within the same tissue 96-98. These observations underscore APA as a highly adaptable mechanism that enables transcriptomes to respond precisely to local cellular environments and functional demands.

## 4. RNA variation at the transcript level

Post-transcriptional RNA modifications constitute a critical layer of gene expression regulation, influencing RNA stability, splicing, nuclear export, translation, and degradation. Broadly, transcripts can be modified in two ways: RNA editing, which alters nucleotide identity 99-101, and chemical modification, which adds chemical groups to RNA without changing the underlying sequence 102. The major types of RNA editing include adenosine-to-inosine (A-to-I) and cytidine-to-uridine (C-to-U) substitutions, as well as insertion/deletion editing events 99,101. In contrast, more than 170 distinct chemical modifications have been identified across mRNAs, rRNAs, tRNAs, and ncRNAs, highlighting their widespread roles in development, immunity, metabolism, and disease, including cancer and neurodegeneration 102. Functionally, RNA editing can recode genetic information and diversify proteomes by altering codons or splice sites 100, whereas chemical modifications primarily regulate RNA fate by modulating structure, stability, localization, and translation efficiency 102. Mechanistically, editing is catalyzed by deaminases or guided insertion/deletion systems 99,101, while chemical modifications rely on diverse writer, reader, and eraser proteins 102. Given several recent comprehensive reviews on RNA editing 99-101, this review will mainly focus on chemical modifications (**Supplemental Table S2**).

Among these, N6-methyladenosine (m6A) is the most abundant and best-characterized internal modification in eukaryotic mRNAs. Installed by the METTL3-METTL14 methyltransferase complex, removed by FTO and ALKBH5 demethylases, and recognized by YTH-domain reader proteins, m6A dynamically regulates mRNA splicing, export, decay, and translation 103-105. Its reversible and site-specific nature underlies its essential functions in embryogenesis, hematopoiesis, neurogenesis, and circadian rhythm regulation, highlighting its central role in gene expression plasticity 106,107.

Additional RNA modifications further diversify transcript-level regulation. N1-methyladenosine (m1A) alters RNA structure and translational fidelity, particularly in tRNAs and rRNAs, while m1A within mRNA 5′ untranslated regions has been linked to enhanced translation under stress 108. 5-methylcytosine (m5C), catalyzed by NSUN and DNMT2 methyltransferases, contributes to mRNA stability, translation efficiency, and cellular stress responses in a transcript-specific manner 109. Pseudouridine (Ψ), the most abundant RNA modification, enhances RNA stability and translational fidelity and has been widely adopted in mRNA therapeutics to reduce immunogenicity and improve translation efficiency 110,111. In parallel, adenosine-to-inosine (A-to-I) RNA editing, mediated by ADAR enzymes, introduces non-templated sequence variation that alters RNA structure and coding potential, expanding proteomic diversity and regulating neuronal plasticity, innate immune tolerance, and antiviral defense, with dysregulation linked to autoimmune and neurodevelopmental disorders 112.

### 4.1. Gene biotypes and RNA modifications

Gene biotypes, functional classifications based on transcriptional output, play a crucial role in shaping the landscape of RNA modifications (**Figure 2C**). Biotypes such as protein-coding genes, pseudogenes, lncRNAs, miRNAs, tRNAs, and rRNAs exhibit distinct modification patterns influenced by their structure, subcellular localization, and function. In turn, RNA modifications regulate the behavior of these biotypes, adding complexity to gene expression.

Protein-coding genes produce mRNAs enriched in m6A, particularly near stop codons and 3′ UTRs. This modification modulates splicing, export, stability, and translation efficiency 113,114. m6A patterns vary across tissues and isoforms, contributing to transcript diversity via alternative splicing and polyadenylation 104. Additional modifications such as N1-methyladenosine (m1A), m5C, and Ψ further refine gene expression, especially under stress or in response to developmental cues 108,110,115. Universal features like 5′ m7G cap, 2′-O-methylation, and poly(A) tail ensure proper mRNA processing and translation 116,117.

Non-coding RNAs exhibit distinct modification profiles. For instance, tRNAs and rRNAs are among the most heavily modified, with dense methylation (e.g., m1A, m5C, and Ψ) being essential for structural stability and translational fidelity 110,115. These modifications prevent mispairing and preserve secondary structure, which is critical for decoding accuracy and ribosome assembly. miRNAs can undergo adenosine-to-inosine (A-to-I) editing by ADAR enzymes, altering their seed sequences and thereby modulating target specificity, thus modulating gene silencing 118.

Long non-coding RNAs often carry m6A modifications that influence their localization, stability, and interactions with protein complexes. For instance, the lncRNA XIST relies on dynamic m6A methylation to mediate X chromosome silencing 119.

Pseudogenes, though often transcriptionally silent, can produce modified RNA transcripts that act as competing endogenous RNAs (ceRNAs), sequestering miRNAs and influencing gene regulation. Modifications like m6A or m5C may stabilize these transcripts, enhancing their regulatory potential 115.

The relationship between RNA modifications and gene biotypes is bidirectional: biotype-specific features shape modification patterns, while modifications fine-tune transcript stability, localization, and translation 104,120. Advances in direct RNA sequencing and crosslinking-immunoprecipitation (CLIP)-based technologies have enabled single-nucleotide resolution mapping, revealing biotype- and stage-specific modification patterns 121,122. Notably, some transcripts, especially under stress, exhibit combinatorial modifications (e.g., m6A and m1A) that cooperatively regulate translation 123.

### 4.2. Roles in development, immunity, metabolism, and cancer

RNA modifications play pivotal roles beyond transcript regulation, influencing development, immune responses, metabolic control, and tumorigenesis. These dynamic epitranscriptomic marks fine-tune gene expression in a tissue- and context-dependent manner.

In embryogenesis, m6A is crucial for stem cell fate and lineage commitment. METTL3-mediated m6A deposition facilitates the degradation of pluripotency-related transcripts, enabling developmental progression. Loss of METTL3 in mouse embryonic stem cells disrupts differentiation and causes developmental arrest, underscoring m6A's essential role in early development 106.

In the immune system, m6A methylation regulates T cell homeostasis, differentiation, and activation by modulating the stability of transcripts encoding key cytokines and transcription factors 124. For instance, METTL3 deletion in CD4+ T cells impairs Th1 and Th17 differentiation, altering inflammatory cytokine production. In innate immunity, m6A also affects dendritic cell maturation and antigen presentation by controlling NF-κB signaling and interferon responses 125. Additionally, A-to-I editing by ADAR1 helps distinguish self from non-self RNA, preventing aberrant activation of antiviral pathways 126.

RNA modifications also regulate metabolic pathways. For example, m6A enhances translation of glycolytic enzymes such as HK2, supporting aerobic glycolysis 127. In brown adipose tissue, METTL3-mediated methylation stabilizes PGC1α transcripts, promoting thermogenesis 128. These mechanisms illustrate how RNA modifications integrate metabolic signaling with gene regulatory networks.

Overall, RNA modifications coordinate a finely tuned system that connects environmental and developmental cues to the post-transcriptional regulation of gene expression. They are central to cellular function and disease, offering novel insights into complex biological processes and potential therapeutic targets.

### 4.3. Therapeutic applications

The dynamic and reversible nature of RNA modifications presents promising opportunities for therapeutic intervention. By targeting RNA-modifying enzymes or incorporating synthetic modifications into therapeutic RNAs, researchers are developing strategies to modulate gene expression, improve RNA stability, and reduce immune detection 129.

In oncology, aberrant mRNA methylation contributes to tumorigenesis, making RNA modification machinery a compelling drug target. Modulating the activity of m6A writers (e.g., METTL3), erasers (e.g., FTO), or readers (e.g., YTHDF1/2) has shown potential in altering tumor progression 130. For example, inhibiting FTO restores m6A levels on tumor suppressor transcripts in acute myeloid leukemia (AML), promoting their degradation and suppressing tumor growth 131. Similarly, depleting YTHDF1 enhances antigen presentation and improves the efficacy of immune checkpoint blockade therapy, suggesting that epitranscriptomic modulation can complement existing immunotherapies 132.

RNA modifications have also been instrumental in advancing mRNA vaccine technology. Incorporating modified nucleosides, such as Ψ and N1-methylpseudouridine (m1Ψ), into synthetic mRNAs enhances translational efficiency and reduces innate immune activation. These modifications stabilize mRNA and prevent recognition by Toll-like receptors and other pattern recognition receptors, thus increasing protein expression and vaccine tolerability 111,133. This strategy was instrumental to the success of mRNA-based COVID-19 vaccines, including Pfizer-BioNTech's BNT162b2 and Moderna's mRNA-1273 133.

In gene therapy, RNA modifications are being harnessed to enhance the performance and safety of RNA-based therapeutics. Chemically modified guide RNAs in CRISPR-Cas systems increase RNA stability and reduce off-target effects 134. Similarly, therapeutic mRNAs for enzyme replacement or regenerative medicine benefit from modifications such as m6A or Ψ, which enhance translation and minimize immunogenicity. These improvements support prolonged protein expression and minimize adverse immune responses, critical factors for clinical success 135.

As our understanding of the epitranscriptome expands, RNA modifications continue to emerge as powerful tools for treating cancer, infectious diseases, genetic disorders, and more.

### 4.4. Detection methods and milestones

The comprehensive elucidation of RNA chemical modifications across the transcriptome has been driven by major advances in both sequencing technologies and computational analysis, enabling increasingly precise, high-throughput, and functionally informative detection of RNA modifications. Early transcriptome-wide mapping efforts established the foundation for linking chemical modifications to gene regulation, while more recent approaches now allow direct, multi-modification detection at single-molecule resolution.

A pivotal milestone was the development of antibody-based enrichment methods such as m6A-seq 136 and MeRIP-seq 137. These approaches use modification-specific antibodies to capture methylated RNA fragments, followed by next-generation sequencing to map modification sites transcriptome-wide. They revealed that N6-methyladenosine (m6A) is widespread, enriched near stop codons and within 3′ UTRs, and dynamically regulated across tissues and conditions. Importantly, these methods demonstrated that RNA modifications are not static marks but play active roles in regulating mRNA stability, splicing, and translation, thereby establishing the field of epitranscriptomics.

A major technological leap came with the introduction of nanopore-based direct RNA sequencing 138, which enables sequencing of full-length native RNA molecules without reverse transcription or amplification. This preserves endogenous chemical modifications and allows their detection through characteristic disruptions in ionic current signals as RNA passes through nanopores. Unlike antibody-based methods, nanopore sequencing provides isoform-level resolution and the ability to detect multiple modification types within individual RNA molecules, offering new insights into how combinations of modifications influence RNA function.

To extract biologically meaningful information from these complex signals, computational frameworks have become essential. For example, Nanocompore 139 uses comparative statistical modeling of nanopore signal distributions between modified and control samples to identify modification sites with high positional accuracy. More recently, TandemMod 140 applies deep learning and transfer learning strategies to simultaneously detect multiple RNA modifications (e.g., m6A, m5C, m7G, Ψ) directly from raw nanopore data. This approach significantly improves sensitivity, scalability, and cross-species applicability, enabling comprehensive profiling of RNA modification landscapes under diverse biological conditions.

Together, these experimental and computational advances have transformed the detection of RNA chemical modifications from enrichment-based snapshots to direct, quantitative, and multi-dimensional measurements. This progression has been critical for uncovering how RNA modifications regulate transcript processing, stability, and translation, ultimately shaping cellular function and disease phenotypes.

### 4.5. Database resources

The rapid expansion of the epitranscriptomics field has led to the creation of several specialized databases that consolidate experimental data, computational predictions, and structural information on RNA modifications. These resources are essential for integrating diverse datasets, generating hypotheses, and guiding functional follow-up experiments.

One of the foundational resources is MODOMICS, which provides a comprehensive catalog of RNA modification pathways, chemical structures, associated enzymes, and biosynthetic routes. It remains a vital reference for researchers investigating the enzymatic machinery behind over 170 known RNA modifications. MODOMICS is especially useful for annotating modifications found in tRNA and rRNA, while also offering insights into their evolutionary conservation and functional implications 141.

Building on this foundation, RMBase expands the scope by integrating transcriptome-wide high-throughput sequencing data. It covers a broader range of RNA species, including mRNA, lncRNA, and circRNA, and combines modification data with cross-linking immunoprecipitation sequencing (CLIP-seq) and other transcriptomic profiles. This enables users to explore RNA-protein interactions, modification motifs, and regulatory networks in greater detail 142.

For researchers focused on m6A, the most prevalent internal mRNA modification, the m6A-Atlas offers a curated, species-diverse database of m6A modification sites derived from MeRIP-seq, miCLIP, and direct RNA sequencing datasets. It includes detailed annotations of modification sites, associated gene ontology terms, and tissue-specific expression patterns, making it a powerful tool for studying the functional roles of m6A in both health and disease 143.

Collectively, these databases are indispensable tools for decoding the complexity of the epitranscriptome. As detection technologies become more sensitive and transcriptomic datasets continue to expand, these resources will play an increasingly important role in integrating multi-omics data and advancing our understanding of RNA-based regulation.

## 5. Conceptual synthesis: Regulated RNA variants as the minimal functional units of genomes

Using more than 18,000 protein-coding genes across 23 cell types from 798 human tissue samples, Reyes and Huber 144 demonstrated that transcriptome diversity is driven primarily by ATS and APA site usage, rather than by AES events. Substantial evidence indicates that 3′ untranslated regions (3′UTRs) harbor a wide array of regulatory cis-acting elements, including adenylate-uridylate (AU-rich) elements (AREs), GU-rich elements (GREs), CU-rich elements (CUREs), CA-rich elements (CAREs), iron-responsive elements (IREs), selenocysteine insertion sequence (SECIS) elements, and others 145. These 3′UTR elements play critical roles in fine-tuning gene expression through interactions with diverse trans-acting factors. Motivated by the central role of transcript termini in gene regulation, we developed whole-transcriptome termini site sequencing (WTTS-seq) to comprehensively capture transcript abundance and usage patterns 146. In this review, we therefore use APA events as a representative framework to illustrate that alternative transcripts—rather than genes themselves—constitute the minimal functional units of genome expression. Specifically, we leverage APA-mediated transcript diversity to address several fundamental biological questions, including why women tend to live longer than men, what causes inbreeding depression, and how cannabis can be leveraged therapeutically to treat obesity.

### 5.1. Why women tend to live longer than men

In humans and most mammals, females consistently outlive males. For example, women live on average 4-7 years longer than men in contemporary populations 147,148. Major hypotheses for this phenomenon include both biological factors—such as enhanced cardiovascular protection associated with higher estrogen levels, stronger immune responses resulting from two X chromosomes, and improved mitochondrial function due to maternal inheritance—and behavioral factors, including lower engagement in risky activities and better health maintenance behaviors in women compared with men 147-151. Here, we propose an RNA-based explanation for female longevity using *Xenopus tropicalis* as a model organism 98. Alternative polyadenylation (APA) events were profiled in embryos at five developmental stages (6, 8, 11, 15, and 28) and in adults at two stages (6 months, young adults; and 18 months, mature adults). Although this study did not directly measure lifespan, it identified robust molecular mechanisms that may underlie sex differences in longevity and resistance to aging.

**Figure 3:**
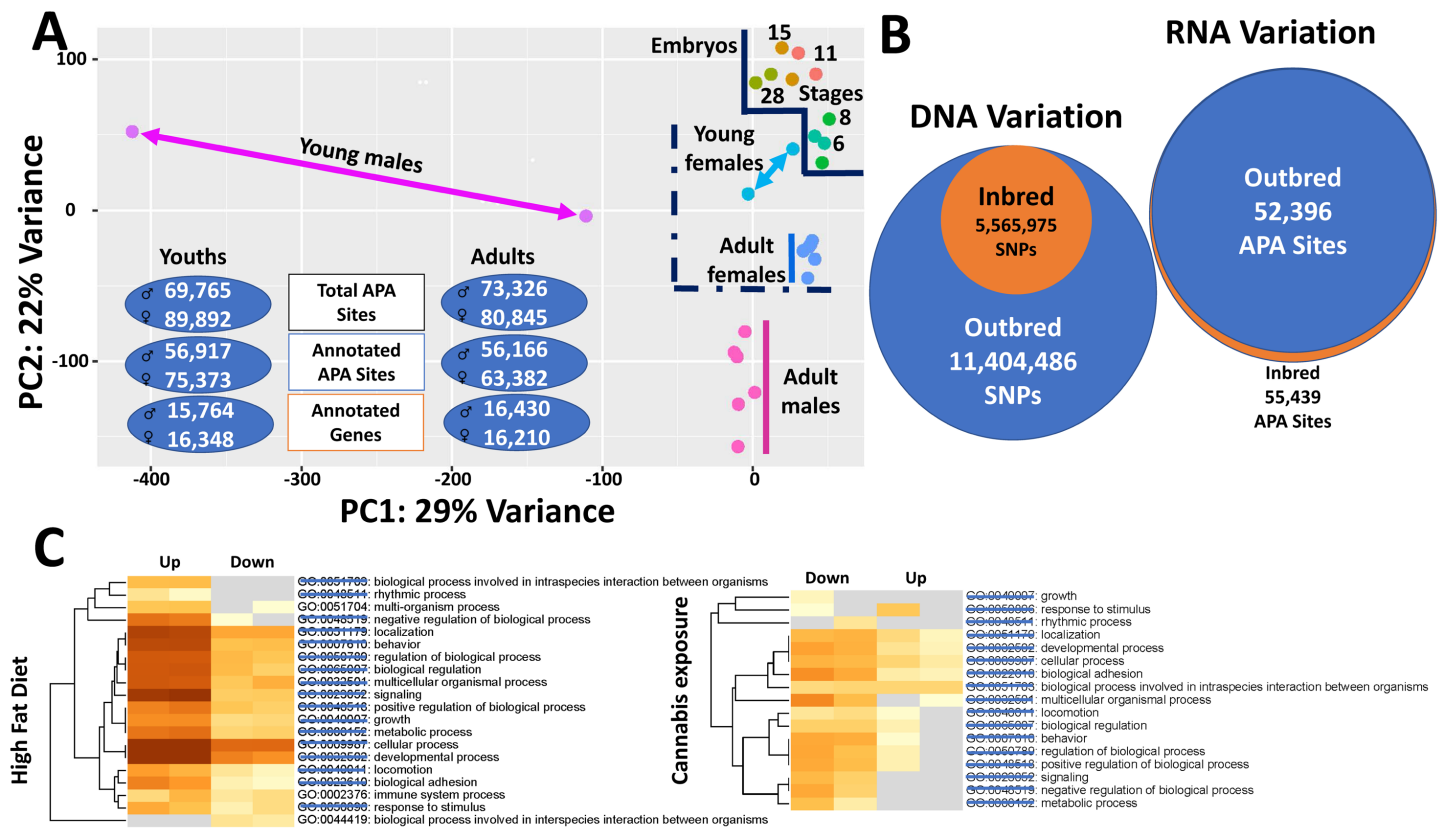
Functional consequences of RNA variation. (A) RNA variation across the lifespan in *X. tropicalis*. Alternative polyadenylation (APA) profiling was performed in embryos, young animals, and adults. Transcriptomic states segregate clearly among embryos, males, and females, with females clustering more closely to embryos than males based on APA profiles. Inter-individual transcriptomic diversity is greater in males than in females, but this diversity decreases from the young to adult stage. Despite this increased variability, males utilize fewer distinct transcripts overall than females. (B) RNA variation in inbred versus outbred populations of swine. Genome purification through inbreeding, resulting in an approximately 50% reduction in single-nucleotide polymorphisms, does not lead to a corresponding decrease in transcript diversity compared with outbred populations. These observations indicate that transcriptome complexity can be maintained independently of underlying DNA sequence variation.(C) RNA variation in response to environmental perturbations in rat. Transcriptomic responses to high-fat diet and cannabis exposure share the majority of affected pathways, with only three pathways unique to one condition. Notably, parental pathway responses are opposite in direction between the two treatments, suggesting that distinct perturbations can converge on similar transcriptomic outcomes through compensatory or antagonistic regulatory mechanisms.

First, we observed that female transcriptomes are more stable than those of males across lifespan stages (**Figure 3A**) 98. In terms of transcriptome diversity, APA usage was more variable in males than in females, particularly at younger ages. Consequently, some males may lack sufficient functional regulatory capacity to cope with physiological challenges, consistent with epidemiological evidence of higher male mortality during early childhood 148. With respect to transcriptome progression, male frogs diverged more substantially from the embryonic transcriptomic state than females, suggesting that females better preserve a more “youth-like” post-transcriptional regulatory profile over time 152. Regarding transcriptome quantity, males utilized significantly fewer APA sites than females. Specifically, young males used 20,127 fewer total APA sites, 18,456 fewer annotated APA sites, and 584 fewer annotated genes compared with young females 98. Greater utilization of genomic regulatory resources in females likely confers increased regulatory plasticity, enabling enhanced adaptive flexibility without requiring genomic expansion or increased regulatory instability 71. Collectively, these findings indicate that males exhibit greater regulatory drift, a known contributor to aging-associated functional decline 71,152.

Second, males and females preferentially utilize different genomic regions to execute APA events 98. Overall, females used 7-8% more exonic APA sites but 8-9% fewer intronic APA sites than males. In addition, young and mature males exhibited 9% and 6% more APA sites, respectively, adjacent to adenosine-rich genomic regions (As or ARSs) than females 98. These differences became more pronounced when differentially expressed APA (DE-APA) sites were compared. In this study, DE-APA sites were classified into three groups: sites commonly used at both young and mature stages, sites specific to young animals, and sites specific to mature animals. Intronic DE-APA sites accounted for 23-45% in males but only 4-6% in females across these three groups. Common DE-APA sites were predominantly distal compared with sex-specific sites in both males (common 40% > mature males 28% > young males 18%) and females (common 58% > young females 43% > mature females 33%). Moreover, a higher proportion of DE-APA sites adjacent to ARSs was observed in males (29-57%) than in females (12-19%). Collectively, these results indicate that females preferentially use exonic and distal APA sites, whereas males predominantly utilize intronic APA sites adjacent to ARSs. Evidence suggests that intronic APA events frequently generate truncated transcripts or unstable, regulatory-shortened RNAs, potentially placing males at a disadvantage relative to females in terms of organismal fitness, health span, and lifespan 148.

Third, we identified sex-specific pathway enrichment patterns that suggest differential allocation of biological resources toward expenditure in males and maintenance in females 98. Pathway enrichment analysis using differentially expressed APA (DE-APA)-associated genes revealed that females were predominantly enriched for pathways involved in cell cycle checkpoints, chromosome organization, DNA repair, peptidyl-lysine modification, RNA localization, cell cycle phase transition, DNA replication, chromosome segregation, microtubule cytoskeleton organization, mRNA processing, and chromatin organization. In contrast, the most highly enriched pathways in males were associated with muscle structure development, actin filament-based processes, muscle system function, drug metabolism, and cell morphogenesis involved in differentiation. Together, these findings indicate distinct molecular “life-history strategies” between the sexes: females prioritize genome stability, transcriptional and post-transcriptional regulation, and cellular maintenance, whereas males emphasize energy-intensive processes related to muscle development and functional output. Such sex-biased investment in somatic maintenance versus physiological expenditure has been widely associated with differences in aging trajectories and survival, with enhanced DNA repair, chromatin regulation, and RNA processing capacity contributing to improved longevity and health span 148,152,153. These molecular signatures are consistent with epidemiological and experimental evidence showing that females, including women, generally exhibit longer lifespans than males, in part due to superior cellular maintenance and reduced cumulative molecular damage over time 153,154.

### 5.2. What causes inbreeding depression

Inbreeding refers to mating between genetically related individuals, a process that increases genome-wide homozygosity and exposes recessive alleles that are typically masked in outbred populations 155,156. A major consequence of inbreeding is inbreeding depression, defined as a reduction in fitness-related traits such as survival, growth, fertility, and disease resistance. Inbreeding depression is widespread across plants, animals, and humans, and poses a serious threat to population viability, particularly in small, isolated, or fragmented populations 156,157.

Two principal hypotheses explain the genetic basis of inbreeding depression. The dominance hypothesis proposes that increased homozygosity unmasks deleterious recessive alleles, thereby reducing fitness 155,157. In contrast, the overdominance hypothesis posits that heterozygotes have superior fitness relative to either homozygote, and that inbreeding disrupts these advantageous heterozygous genotypes 155,158. Empirical evidence across taxa indicates that the dominance hypothesis accounts for the majority of inbreeding depression, although overdominance may contribute in specific loci or traits 156,157. Importantly, both hypotheses are largely gene-centric and have historically focused on coding variation, with limited consideration of post-transcriptional regulatory mechanisms.

Using both inbred (16 generations) and outbred Luchuan pigs, Han and coworkers 159 integrated whole-genome sequencing with transcriptome-wide alternative polyadenylation (APA) profiling to investigate molecular mechanisms underlying inbreeding depression (**Figure 3B**). At the genomic level, outbred pigs harbored 11,404,486 single-nucleotide polymorphisms (SNPs), whereas only 5,565,975 SNPs remained in the inbred population, reflecting extensive loss of genetic diversity. Correspondingly, inbred individuals exhibited 4,511 runs of homozygosity (ROH), including 3,719 ROH shorter than 1 Mb, 738 ranging from 1-2 Mb, and 54 exceeding 2 Mb in length 159. Despite this substantial purging of genomic polymorphisms, inbreeding did not result in a proportional reduction in the overall number of APA events, suggesting that APA regulation is not passively constrained by genetic diversity loss.

Three tissues—liver, leg muscle, and submandibular lymph nodes—were profiled for APA usage in both inbred and outbred groups (15 individuals per group). At the whole-transcriptome level, 63,282 APA sites were identified, with 55,439 and 52,396 sites expressed in inbred and outbred pigs, respectively. Thus, 16 generations of inbreeding did not reduce APA site usage; instead, inbred animals exhibited more than 3,000 additional APA sites compared with outbred counterparts. At the tissue level, inbred animals expressed 35,010 APA sites in liver, 30,489 in leg muscle, and 43,146 in lymph nodes, whereas outbred animals expressed 35,525, 35,056, and 36,877 sites, respectively. Notably, inbred animals displayed markedly greater tissue-to-tissue variability in APA usage (12,657 sites) compared with outbred animals (1,821 sites), indicating pronounced dysregulation of tissue-specific APA programs.

While the global number of APA sites was maintained or even increased, genes associated with differential APA usage showed a strong bias toward downregulation in inbred animals. In liver, 336 genes were downregulated versus 257 upregulated; in muscle, 1,907 versus 367; and in lymph nodes, 1,885 versus 947 159. Functional enrichment analyses revealed that inbred pigs exhibited compromised liver functions related to lipid metabolism, small-molecule and nucleotide metabolism, metal ion responses, and hydrolase activity. In muscle, downregulated APA-associated genes were enriched for muscle structural components, skeletal muscle contraction, muscle development, and—critically—mRNA metabolism and RNA splicing. In lymph nodes, affected genes were enriched for immune regulation, leukocyte and lymphocyte differentiation, and transforming growth factor-β receptor signaling 19. Together, these results indicate that inbreeding depression is associated not merely with gene loss or reduced expression, but with widespread disruption of post-transcriptional regulatory architecture, particularly APA-mediated control of gene output.

Based on the evidence presented, we propose that inbreeding depression is driven largely by instability in alternative transcript regulation, rather than solely by the expression of deleterious coding alleles. Long-term inbreeding increases genome-wide homozygosity at both cis-regulatory elements and trans-acting RNA regulatory factors, reducing the buffering capacity that normally stabilizes alternative transcript choices, including alternative polyadenylation, splicing, and transcription termination 71,155. As a result, inbred genomes exhibit increased regulatory noise, loss of tissue-specific coordination, and biased usage of suboptimal transcript isoforms, leading to reduced mRNA stability, impaired translation, and weakened gene output despite the preservation of gene number and overall transcript abundance 71,155. The enrichment of alternative transcript changes in genes governing muscle development, immune function, metabolism, and RNA processing itself suggests that this regulatory instability can propagate through feed-forward mechanisms, amplifying physiological dysfunction and contributing to reduced fitness 156,159. This model extends classical dominance-based explanations of inbreeding depression by identifying disrupted alternative transcript regulation as an independent and systems-level mechanism underlying fitness decline.

### 5.3. How cannabis can be leveraged therapeutically to treat obesity

Obesity and cannabis use are both associated with adverse health outcomes, yet epidemiological and experimental studies have paradoxically suggested that cannabis exposure may exert protective effects against obesity. Population-based studies report lower body mass index (BMI), reduced prevalence of obesity, and improved metabolic profiles among cannabis users compared with non-users, despite increased caloric intake in some cases 160-162. Pharmacological studies further indicate that cannabinoids can modulate appetite, energy balance, lipid metabolism, and insulin sensitivity through central and peripheral mechanisms 163,164. However, these effects are context dependent and incompletely understood, particularly at the level of transcript regulation in the central nervous system. Thus, while cannabis has emerged as a potential therapeutic or modulatory agent for obesity, the molecular mechanisms underlying its effects remain poorly defined.

Using APA profiling in the rat hypothalamus, we examined transcriptomic responses to high-fat diet-induced obesity and to cannabis exposure in two independent studies (**Figure 3B**) 165,166. Obesity was associated with widespread APA activation, with 763 differentially expressed APA sites, the majority of which were upregulated, consistent with transcriptional and post-transcriptional activation of metabolic and neuroendocrine pathways 165. In contrast, cannabis exposure resulted in a predominance of APA downregulation, with 243 of 309 differentially expressed APA sites reduced relative to controls 166. Pathway reanalysis revealed that 17 canonical pathways—including neuroactive ligand-receptor interaction, insulin signaling, MAPK signaling, cAMP signaling, synaptic transmission, and circadian entrainment—were fully shared between the two conditions but regulated in opposite directions. Strikingly, only 103 genes overlapped between the datasets, indicating that cannabis does not reverse obesity by targeting identical genes, but instead restores pathway-level balance through alternative transcript usage. These findings suggest that negative physiological states can be mitigated not by gene-specific correction, but by counteracting dysregulated regulatory networks—a principle consistent with systems-level models of disease modulation.

## 6. Conclusion

A persistent challenge in genetics is reconciling the limited number of genes with the extraordinary breadth of phenotypic diversity observed across organisms, tissues, and conditions. The evidence reviewed here supports a unifying view: regulated RNA variants constitute the minimal functional units through which genomes achieve both efficiency and complexity. Through layered and combinatorial processes—including gene biotype diversification, alternative transcriptional and post-transcriptional processing, RNA editing, and chemical modification—organisms expand regulatory capacity without proportional expansion of genomic sequence.

**Figure 4:**
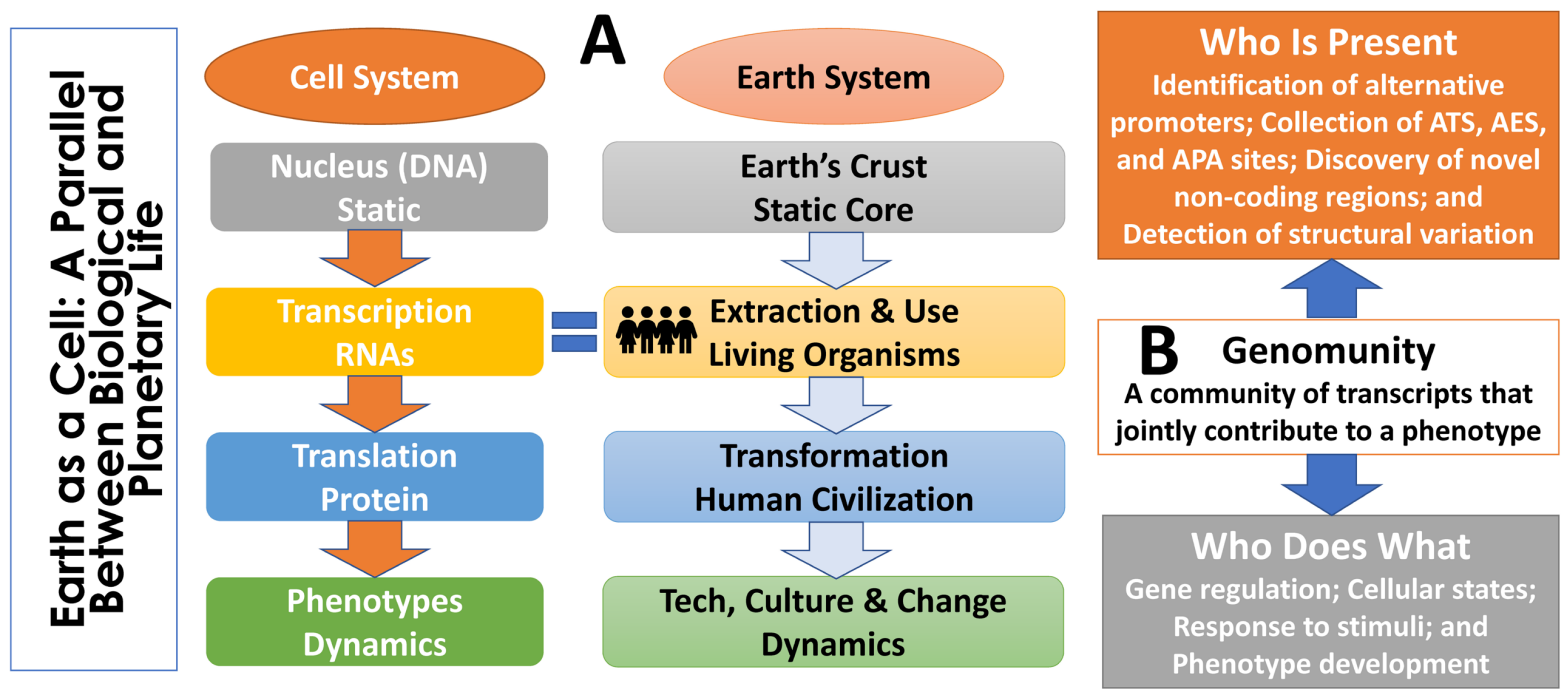
The concept of genomunity. (A) Analogy between biological and planetary life. The Earth's physical structure provides a stable but largely inert foundation; it becomes dynamic and expressive only through the activity of living organisms. A similar division exists within the cell: DNA serves as a stable blueprint, whereas RNA, proteins, and metabolites actively interpret and implement genomic information. In this analogy, biological function arises not from static structure alone but from dynamic agents—*RNA are people too*—that transform latent potential into observable outcomes. (B) Immediate action plan for defining genomunities. Progress requires coordinated efforts along two axes: identifying *who is present* (comprehensive characterization of RNA variants and transcript diversity) and determining *who does what* (functional dissection of how RNA variants and their communities influence gene regulation, cellular states, response to stimuli and phenotypes).

As illustrated in Figure 4, this relationship mirrors the distinction between planetary structure and planetary life. The Earth's rocks, minerals, and physical geography provide a stable substrate rich in potential but are, by themselves, largely inert. It is the collective activity of living organisms that renders the planet dynamic, adaptive, and expressive. An analogous division exists within the cell: DNA provides a stable and heritable specification of biological potential, whereas RNA and its downstream molecular effectors actively interpret and deploy that information. In this sense, while DNA defines the genomic landscape, RNA are people too—the agents that transform static information into biological activity.

To formalize this perspective, we introduce the concept of a *genomunity*, defined as a community of RNA transcripts whose coordinated activity gives rise to a phenotype. Genomunities provide a conceptual bridge between genotype and phenotype by shifting emphasis from individual genes to collective RNA programs. They naturally accommodate non-additive interactions among RNA variants, capture context specificity across development, tissue type, sex, and environment, and help explain phenotypic convergence arising from distinct genetic perturbations.

Advancing the genomunity framework requires immediate progress along two complementary axes. First, defining *who is present* demands comprehensive identification of RNA diversity, including systematic mapping of alternative promoters; cataloging alternative transcription start, exon skipping, and polyadenylation sites; discovery of previously unannotated coding and non-coding transcripts; and detection of structural variation that reshapes transcript architecture. Second, determining *who does what* requires quantitative and mechanistic assessment of how RNA variants influence gene function, including effects on transcript abundance, stability, localization, translational output, protein structure, and epigenetic regulation. Importantly, these effects are expected to emerge at the level of transcript communities rather than individual RNA species.

Taken together, this framework positions RNA variation not as a secondary regulatory layer but as a central organizing principle of genome function. By enabling limited genomic information to be flexibly interpreted across contexts, RNA variation underlies both genomic efficiency and phenotypic complexity. As transcriptome-resolved, isoform-aware, and modification-sensitive technologies continue to mature, incorporating genomunity-level analyses into genetic, evolutionary, and disease models will be essential for a mechanistic understanding of how biological systems generate complexity, adapt, and fail.

## Supplementary Material

Supplementary tables.
