# Supplementary material for: RNA variation as the driver of genomic efficiency and phenotypic complexity
Source: Int J Biol Sci. 2026 May 29;22(11):5969–87. doi: 10.7150/ijbs.135200 (PMC13282790; doi:10.7150/ijbs.135200)
Supplement: Supplementary file 1 — Supplementary tables. [file ijbsv22p5969s1.pdf]

Supplemental Table S1. RNA Types: Coding and Non-Coding Overview

RNA Type	Length	Origin / Location	Primary Function	Category	Key Role
**Messenger RNA (mRNA)**	Variable (~500–10,000+ nt)	Nucleus → cytoplasm	Encodes protein sequence	Coding	Template for protein synthesis at ribosomes
**Precursor mRNA (pre-mRNA)**	Variable	Nucleus	Intermediate for mRNA	Coding (precursor)	Contains introns; undergoes splicing, capping, and polyadenylation
**Ribosomal RNA (rRNA)**	Variable (~120–5,000 nt)	Nucleolus	Protein synthesis	Housekeeping ncRNA	Forms ribosomes; catalyzes peptide bond formation
**Transfer RNA (tRNA)**	~70–90 nt	Nucleus → cytoplasm	Amino acid transport & translation	Housekeeping ncRNA	Delivers amino acids during translation
**Small Nuclear RNA (snRNA)**	~150 nt	Nucleus	Splicing and RNA regulation	Both (HK & Reg ncRNA)	Spliceosome component; modulates splicing
**Small Nucleolar RNA (snoRNA)**	~60–400 nt	Nucleolus	RNA modification	Both (HK & Reg ncRNA)	Modifies rRNA, tRNA, snRNA; affects expression
**RNase P RNA**	~350–400 nt	Nucleus	tRNA maturation	Housekeeping ncRNA	Processes pre-tRNA to mature form
**RNase MRP RNA**	~270 nt	Nucleolus, mitochondria	rRNA processing & mtDNA replication	Housekeeping ncRNA	Cleaves rRNA precursors, supports mtDNA replication
**microRNA (miRNA)**	~22 nt	Nucleus → cytoplasm	Post-transcriptional regulation	Regulatory ncRNA	Represses or degrades mRNA
**Small Interfering RNA (siRNA)**	~21–25 nt	From dsRNA	Gene silencing	Regulatory ncRNA	Targets mRNA for degradation via RISC
**Piwi-Interacting RNA (piRNA)**	~26–31 nt	Germline cells	Transposon silencing	Regulatory ncRNA	Maintains genome integrity in germline

**Circular RNA (circRNA)**	Variable	Back-spliced exons	miRNA sponge & protein interaction	Regulatory ncRNA	Sequesters miRNAs; binds transcription machinery
**Enhancer RNA (eRNA)**	Variable	Enhancer regions	Transcriptional regulation	Regulatory ncRNA	Modulates gene transcription
**tRNA-derived Fragments (tRF/tsRNA)**	~14–40 nt	tRNA cleavage	Translation repression	Regulatory ncRNA	Controls stress response, signaling
**moRNA**	~19–22 nt	miRNA flanks	Putative regulation	Regulatory ncRNA	Possible gene regulation role
**shRNA-derived miRNA / milRNA**	~21–24 nt	Hairpin precursors	Post-transcriptional silencing	Regulatory ncRNA	Functions like miRNAs
**PASR**	~20–90 nt	Promoter regions	Transcriptional regulation	Regulatory ncRNA	Modulates transcription initiation
**TSSa-RNA**	~20–90 nt	TSS regions	Transcriptional control	Regulatory ncRNA	Linked to gene activation/inhibition
**TASR**	~20–90 nt	Gene termini	Transcriptional control	Regulatory ncRNA	May affect transcription elongation/termination
**tiRNA**	~20–90 nt	TSS regions	Initiation regulation	Regulatory ncRNA	Supports RNA pol recruitment
**spliRNA**	~20–30 nt	Exon-intron junctions	Splicing regulation	Regulatory ncRNA	Guides splice site selection
**snoRNA-derived RNA (sdRNA)**	~20–30 nt	snoRNA processing	Translation & stability regulation	Regulatory ncRNA	Post-transcriptional gene regulation
**qiRNA**	~20–21 nt	DNA damage-induced	Genome integrity	Regulatory ncRNA	Represses expression after DNA damage
**Small Vault RNA (svRNA)**	~23 nt	Vault RNAs	Drug resistance & regulation	Regulatory ncRNA	Involved in multidrug resistance pathways

Supplemental S2. RNA Variation Classification

Variance Classification	RNA Variance
**Assorted RNA Variance**<br>○ **RNA variance based on gene biotypes producing diverse RNA products.**<br><br>**(Antisense is both assorted and processed)**	○ Alternative Splicing: Variance in exon inclusion or exclusion leads to multiple RNA isoforms.<br>○ Exon Skipping: A form of alternative splicing where entire exons are skipped.<br>○ Intron Retention: A splicing variant where introns are retained within the transcript.<br>○ Mutually Exclusive Exons: One of two (or more) exons is included in the final transcript.<br>○ Trans-splicing: Exons from two separate pre-mRNA molecules are joined together.<br>○ Tandem UTR Variance: Multiple UTR regions are utilized, affecting transcript stability and translation efficiency.<br>○ Bidirectional Transcription: Transcription initiated from opposite strands of DNA produces overlapping RNA products.<br>○ Exon Duplication: Additional copies of an exon are included in the mRNA.<br>○ Exon Deletion: Entire exons are deleted from a transcript during splicing.<br>○ Pseudogenes: non-functional copies of genes that arise from gene duplication or retrotransposition events.
**Processed RNA Variance**<br>○ **Productions of different RNA lengths and isoforms from a single gene.**	○ Alternative Transcription Start Sites (TSS): Multiple initiation points lead to diverse RNA isoforms.<br>○ Alternative Polyadenylation (APA): Variance in the poly(A) tail addition site, altering the 3' UTRs.<br>○ Alternative 5' Splice Site: A different splice donor site is used.<br>○ Alternative 3' Splice Site: A different splice acceptor site is used.<br>○ Tandem UTR Variance: Multiple UTR regions are utilized, affecting transcript stability and translation efficiency.<br>○ Bidirectional Transcription: Transcription initiated from opposite strands of DNA produces overlapping RNA products.<br>○ Cryptic Splicing: The use of non-canonical splice sites, often leading to dysfunctional proteins.<br>○ mRNA Length Variance: Different transcript lengths arise from alternative TSS, APA, or splicing.<br>○ Full-Length Transcripts: The complete version of the RNA transcript, as opposed to truncated versions.<br>○ Truncated Transcripts: Shorter RNA transcripts due to premature termination or incomplete processing.

	o Incompletely Spliced Transcripts: RNA molecules that contain retained introns.<br>o Internal Transcription Start Sites: Initiation of transcription from within the gene, producing shorter RNA variants.<br>o Multiple Isoforms from a Single Gene: The production of multiple RNA isoforms with distinct lengths and functions.<br>o Nested Transcripts: One transcript is located within the intronic region of another gene.<br>o Read-through Transcription: RNA polymerase continues transcription past the normal stop signal, creating extended RNA molecules.<br>o Fusion Transcripts: Two previously separate genes are transcribed as one RNA molecule, usually due to chromosomal rearrangements.<br>o Overlapping Transcripts: Two transcripts from different genes overlap on the genome.
**Modified RNA Variance**<br>o **Post-transcriptional modifications that affect RNA molecules without altering RNA sequence**	o N6-Methyladenosine (m6A): A common modification that impacts RNA stability and splicing.<br>o 5-Methylcytosine (m5C): A methylation of cytosine residues in RNA, affecting its function.<br>o Pseudouridylation: The conversion of uridine to pseudouridine, which can alter RNA secondary structure.<br>o Adenosine-to-Inosine (A-to-I) Editing: Editing that changes adenosine to inosine, affecting RNA base pairing.<br>o N1-Methyladenosine (m1A): A modification that affects tRNA and mRNA structure and function.<br>o 2'-O-Methylation: A modification of the ribose sugar that can impact RNA stability and function.<br>o Cap-Dependent Modifications (e.g., 7-methylguanosine cap): The 5’ cap structure is modified to enhance stability and translation.<br>o RNA Acetylation (ac4C): Acetylation of cytidine residues, influencing mRNA translation.<br>o Uridylation: The addition of uridine residues to the 3' end of RNA, often leading to degradation.<br>o RNA Methylation (m5U): Methylation of uridine residues, altering the stability of tRNA.<br>o N7-Methylguanosine (m7G): A modification found in tRNAs and mRNAs influencing translation efficiency.<br>o 2-Thiouridine (s2U): A sulfur-modified uridine affecting tRNA decoding efficiency.<br>o Queuosine Modification: A tRNA modification that impacts accuracy in translation.<br>o m6Am Modification: A modification near the 5' cap of mRNA that can influence stability and translation.

	○ <u>RNA Glycosylation:</u> The addition of sugar molecules to RNA, potentially impacting its function.<br>○ <u>tRNA Modification</u> (e.g., m1G, m3C, t6A): Modifications affecting tRNA stability, folding, and decoding.<br>○ <u>Y-base Modification in tRNA:</u> A hypermodified base found in certain tRNAs that improves translation efficiency.<br>○ <u>RNA Oxidation (8-oxoG):</u> Oxidative modifications that can alter RNA function or lead to degradation.<br>○ <u>RNA Phosphorylation:</u> Phosphorylation of RNA molecules, potentially influencing their activity in stress responses.
